# Supplementary material for: Facile Purification and Use of Tobamoviral Nanocarriers for Antibody-Mediated Display of a Two-Enzyme System
Source: Viruses. 2023 Sep 19;15(9):1951. doi: 10.3390/v15091951 (PMC10536799; doi:10.3390/v15091951)
Supplement: Supplementary file 1 [file viruses-15-01951-s001.zip › viruses-2568331-supplementary.pdf]

viruses

MDPI

Supplementary Data

Facile purification and use of tobamoviral nanocarriers for antibody-mediated display of a two-enzyme system

Tim Wendlandt [a]; Claudia Koch [a], Beate Britz, Anke Liedek, Nora Schmidt, Stefan Werner, Yuri Gleba, Farnoosh Vahidpour, Melanie Welden, Arshak Poghossian, Michael J. Schöning, Fabian Eber, Holger Jeske [b] and Christina Wege [b]*

[a,b] These authors contributed equally to the work.
* Correspondence: Christina Wege, Research Unit Molecular and Synthetic Plant Virology, Institute of Biomaterials and Biomolecular Systems, University of Stuttgart, 70569 Stuttgart, Germany, email: christina.wege@bio.uni-stuttgart.de

Supplementary Figures 1-3, corresponding to the following Results sections:

3.1. TVCV$_{WT}$ and TVCV$_{PA}$ production, isolation and characterization

3.1.2. A standard tobamovirus purification procedure via stepwise enrichment needs adaptation for TVCV$_{PA}$ and yields the expected shortened particles - and:

3.1.3. Virus isolation from crude precipitates by selective inverse PEG-sucrose solubility gradients reduces effort and improves particle integrity

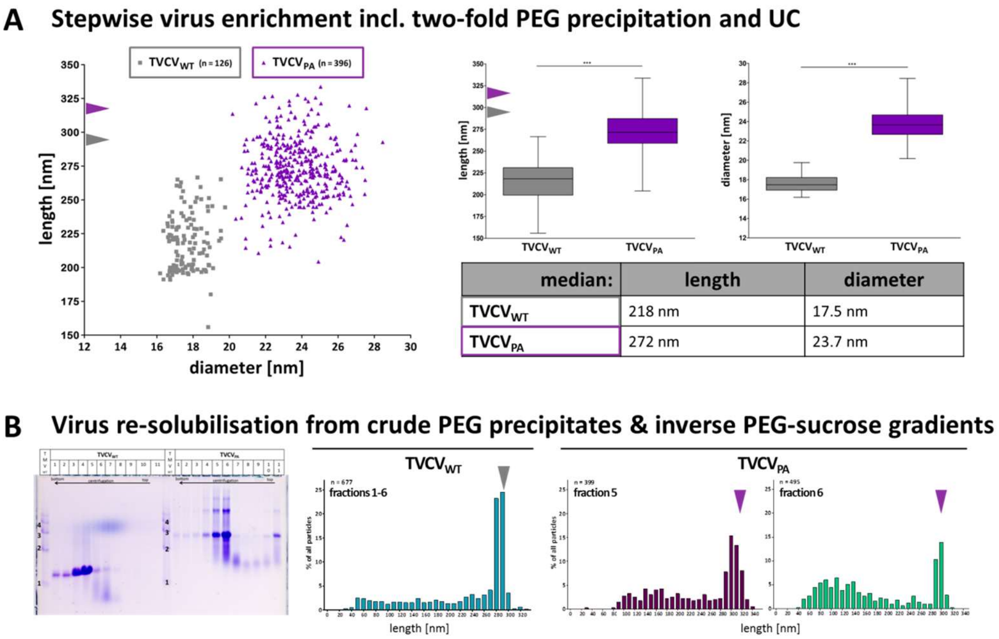

Figure S1. Analyses of virion lengths resulting from two alternative isolation procedures (as indicated). TEM images of randomly selected grid areas with TVCV$_{WT}$ or TVCV$_{PA}$ particles from typical experiments were analyzed by software ImageJ. **A)** After purification through standard stepwise procedures, virion length and diameter distributions in the final preparations were compared. Left: Scatterplots show TVCV$_{WT}$ ($n = 126$) and TVCV$_{PA}$ ($n = 396$) particles in regard to length and diameter. Right: Boxplots of lengths (left) and diameters (right) of TVCV$_{WT}$ and TVCV$_{PA}$ and table showing the median values obtained (lines: median values, box boundaries: 25/75 % quartiles, whiskers: 100 % percentiles). Statistical analyses were performed using the non-parametric Mann–Whitney Rank Sum Test. A p value of less than 0.05 was considered to be significant (* $P < 0.05$; ** $P < 0.01$; *** $P < 0.001$). Arrows: expected particle lengths. **B)** After purification via inverse PEG solubility gradients, particles were analyzed by native agarose gels (left) and TEM (right). In TEM image analysis, the virions corresponding to the fractions of highest purity were attributed to 10 nm length classes. For TVCV$_{PA}$, length distributions in fractions 5 and 6 are shown separately. Arrows indicating the expected particle lengths demonstrate the substantially increased particle integrity obtained through this purification protocol.

3.4. A two enzyme-cascade of GOx and HRP installed on TVCV_{PA} through capture of a single antibody-conjugate: initial binding tests for GFP through anti-GFP IgGs

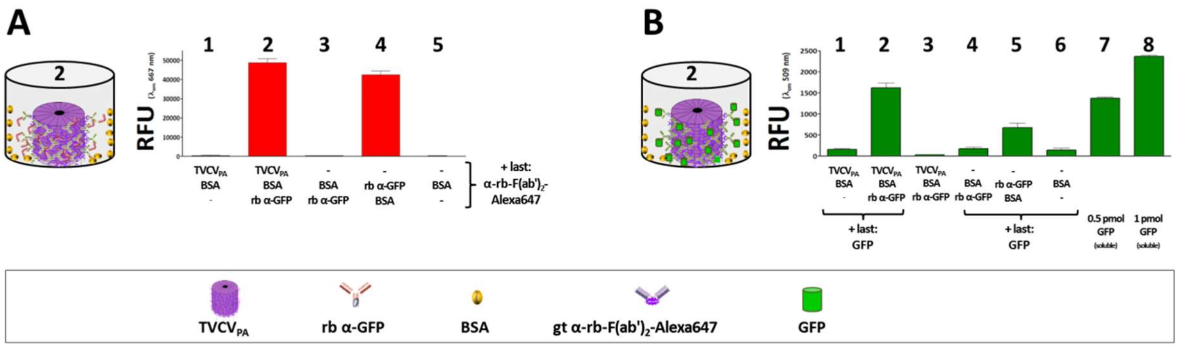

Figure S2. Antibody-mediated immobilization of GFP on TVCV$_{PA}$ particles. A) Scheme: Adapter-(TVCV$_{PA}$)-coated and empty plate wells were subjected to the serial treatments indicated below the diagram (scheme left: layout 2). Binding of rabbit anti-(α)-GFP IgGs was detected by fluorescent secondary F(ab')$_2$-fragments (goat F(ab')$_2$-Alexa647). Fluorescence was determined spectrophotometrically at λ_{Ex} = 630 nm; λ_{Em} = 667 nm. **B)** Layouts as in (A) and additional controls as indicated were tested for binding of GFP from solution. Captured GFP was detected at λ_{Ex} = 470 nm; λ_{Em} = 509 nm. The application of TVCV$_{PA}$ adapters presenting rabbit anti-GFP IgGs yielded an immobilization of around 0.7 pmol GFP/well [column 2], compared to ≈ 0.25 pmol in the corresponding layout lacking TVCV$_{PA}$ [column 5].

3.4. A two enzyme-cascade of GOx and HRP installed on TVCV$_{PA}$ through capture of a single antibody-conjugate

3.4.2. TVCV$_{PA}$-displayed HRP-conjugated antiGOx-IgGs tether both enzymes into a durable cooperating system

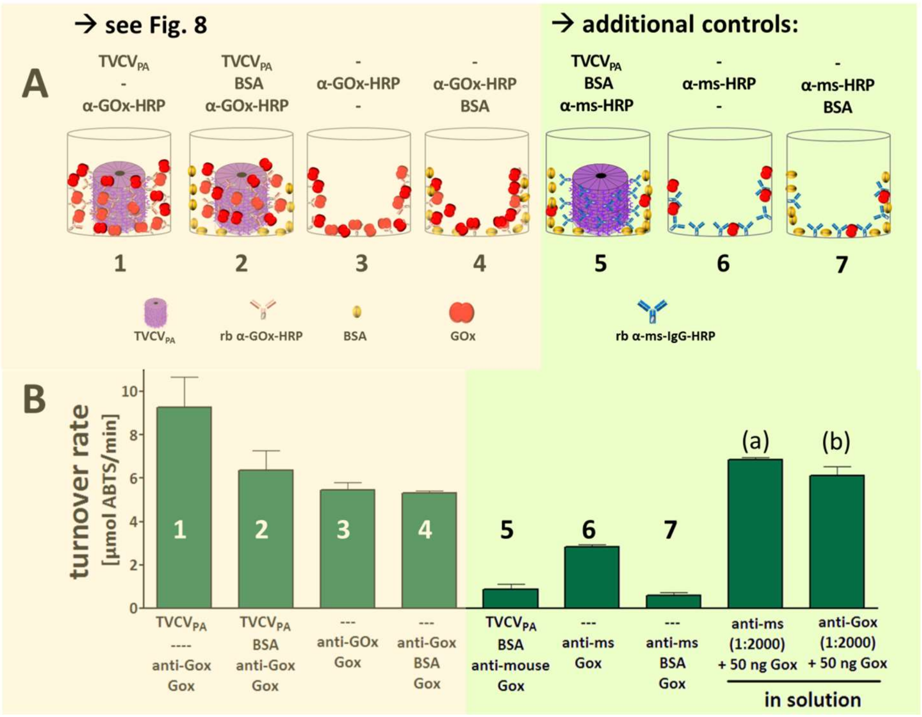

Figure S3 - extension of Fig. 8: Antibody-mediated immobilization of the bi-enzyme cascade GOx/HRP in microtiter plates with or without TVCV$_{PA}$ particles and enzyme activities - controls lacking GOx-specific IgGs. A) Schemes of layouts, and **B)** corresponding ABTS turnover rates. Yellow (left) part: see Fig. 8. Green (right) part: Layouts 5-7: control treatments using rabbit anti-mouse-HRP as non-GOx-directed IgG. **B)** in addition (a)/(b): Enzymatic conversion rates of GOx/antibody mixtures in solution.

See main text for details.
